# Fluid-assisted grain size reduction leads to strain localization in oceanic transform faults

**DOI:** 10.1038/s41467-023-39556-5

**Published:** 2023-07-10

**Authors:** Manon Bickert, Mary-Alix Kaczmarek, Daniele Brunelli, Marcia Maia, Thomas F. C. Campos, Susanna E. Sichel

**Affiliations:** 1 Geo-Ocean, Univ Brest, CNRS, IFREMER, UMR6538, F-29280 Plouzané, France; 2 Dipartimento di Scienze Chimiche e Geologiche, Università di Modena e Reggio Emilia, Modena, Italy; 3 Géosciences Environnement Toulouse (GET), CNRS-CNES-IRD-Université Toulouse III, Observatoire Midi Pyrénées, 14 avenue E. Belin, 31400 Toulouse, France; 4 IGAG-CNR, Istituto di Geologia Ambientale e Geoingegneria, Rome, Italy; 5 Department of Geology, Rio Grande do Norte Federal University, Natal, Rio Grande do Norte Brazil; 6 Department of Geology and Geophysics, Federal Fluminense University, Niteroi, Rio de Janeiro Brazil

**Keywords:** Petrology, Tectonics, Geodynamics

## Abstract

Oceanic Transform Faults are major plate boundaries representing the most seismogenic part of the mid ocean ridge system. Nonetheless, their structure and deformation mechanisms at depth are largely unknown due to rare exposures of deep sections. Here we study the mineral fabric of deformed mantle peridotites - ultramafic mylonites - collected from the transpressive Atobá ridge, along the northern fault of the St. Paul transform system in the Equatorial Atlantic Ocean. We show that, at pressure and temperature conditions of the lower oceanic lithosphere, the dominant deformation mechanism is fluid-assisted dissolution-precipitation creep. Grain size reduction during deformation is enhanced by dissolution of coarser pyroxene grains in presence of fluid and contextual precipitation of small interstitial ones, leading to strain localization at lower stresses than dislocation creep. This mechanism potentially represents the dominant weakening factor in the oceanic lithosphere and a main driver for the onset and maintenance of oceanic transform faults.

## Abstract (web summary)

Deformed mantle rocks exhumed by oceanic transform faults recorded fluid-assisted ductile deformation at high temperatures and pressures, corresponding to the root of the fault. This deformation mechanism controls deep faulting at plate boundaries.

## Introduction

Oceanic Transform Faults (OTFs) are strike-slip boundaries sub-parallel to the plate spreading direction that offset Mid Ocean Ridges (MORs). Their cumulate length represents more than 40% of the global MOR system. Strike-slip motion along these faults generates large earthquakes (*M* > 7), globally releasing 10 to 20 times more seismic energy than that released at the spreading sections of the MOR^1,2^. The downward extent of seismicity along the OTFs is an indirect proxy for the thermal regime constraining the depth of the brittle lithosphere^3^. The Brittle-Ductile Transition (BDT) in the oceanic lithosphere is assumed to correspond to the 600 °C isotherm under normal stress conditions^1,4^. Yet, recent petrological and seismic studies on peridotite mylonites from OTFs showed that brittle deformation can locally extend to temperatures as high as 1000 °C at peak stress conditions^5–8^.

Pervasive shearing at high pressure (>0.6 GPa) and high temperature (>750 °C) generates mylonites and ultramylonites, i.e. strongly foliated, very finely grained metamorphic rocks^5,6,9–12^, representing a rare occurrence on the seafloor. The deformation in the ductile region involves olivine-rich mantle-derived lithologies, containing variable amounts of pyroxene, spinel and occasional amphibole, here grouped under the general term peridotite. These rocks are key to provide constraints on the fault slip mechanics of major lithospheric-sized strike-slip plate boundaries, dramatically affecting densely populated regions such as the Caribbean belt, San Andreas and the northern Anatolian fault systems.

Grain size reduction during mylonitization results in significant decrease of rock viscosity, enabling permanent weakening at large scale and strain localization at depths^13,14^. This process often marks a transition in the creep mechanism from dislocation to diffusion, grain size sensitive creep^10,13–15^. Fluid circulation may drive grain size reduction, as reported in deformed rocks experiencing melt-rock reactions^16–18^, fluid addition^5,19,20^, or metamorphic equilibration to new P-T conditions^21,22^. In all these studies, fluid-assisted grain size reduction leads to very fine-grained well mixed polymineralic assemblages.

**Fig. 1:**
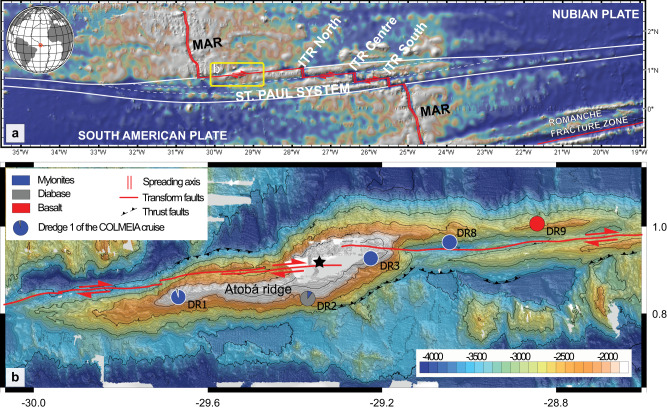
Bathymetric map of the St. Paul Transform System and detail of the Atobá Ridge. **a** Map of the entire St. Paul transform system (delimited by thick white lines), composed by three intra-transform ridge (ITR) segments: ITR North, Centre and South, respectively. MAR: Mid-Atlantic Ridge. The inactive part of the intra-transform faults is marked by white dashed lines. The globe showing the map location is made using GMT software^61^. The yellow box shows the detail of the Atobá Ridge (**b**) located above the northern fracture zone of the St. Paul Transform System. **b** The black star indicates the positions of the St. Paul and St. Peter archipelago. Pizza plots show the relative amounts of rocks recovered by dredging during COLMEIA cruise^23,26^. Selected samples are from the dredged sites highlighted by white contours. Maps are generated from shipborne bathymetry data acquired during COLMEIA cruise^23,26^.

In this work we present new petrological and microstructural data of peridotite mylonites and ultramylonites from the Atobá Ridge, in the St. Paul Transform System (Fig. 1). We show how high temperature fluid-assisted dissolution-precipitation creep is the main process of grain size reduction acting at the roots of oceanic transform faults. This process is marked by a crystallographic preferred orientation of orthopyroxene mimicking the orientation of olivine. We propose fluid-assisted dissolution-precipitation creep as the dominant mechanism to localize strain in the oceanic lithosphere.

## Results

### The Atobá ridge: a tectonic window on a fracture zone

The St. Paul Transform System (TS) is one of the major structures of the global oceanic ridge system, located at 1°N in the Equatorial Atlantic, and offsetting the Mid-Atlantic Ridge (MAR) by about 630 km^23,24^. Four active OTFs oriented E-W dissect the MAR axis in three short intra-transform ridge segments^23–25^ (Fig. 1A). The northern transform fault of the St. Paul TS hosts a major transpressive complex^23^, culminating in the St. Paul and St. Peter islets (Fig. 1B). The islets are the emerged part of a large submarine sigmoidal structure, the Atobá Ridge, a regional-sized push-up ridge raising by 3500 m above the seafloor^23,24^. Rocks dredged on the north flank of the Atobá ridge during the COLMEIA cruise^23,26^ (Fig. 1B; Supplementary Table S1) are mostly ultramafic mylonites and ultramylonites, closely resembling those outcropping on the St. Peter and Paul’s archipelago and first described by Darwin in 1832 during the Beagle expedition^24,25,27–30^. These rocks are strongly deformed, characterized by a layered fine-grained matrix representing 40-90% of the sample (protomylonite to mylonite) to >90% in the ultramylonites, the rest being porphyroclasts (i.e. relics of the initial grains). Atobá ridge mylonites and ultramylonites are interpreted as deriving from a peridotitic protolith that experienced high temperature/high pressure ductile deformation then exhumed by transpressive thrusting along a positive flower structure^23,25,27,28,31^. Hence, the structure of the Atobá ridge represents a tectonic window, giving a unique opportunity to access deep-seated rocks from the roots of the fracture zone.

### Mineralogy and microstructures of St. Paul sheared ultramafics

**Fig. 2:**
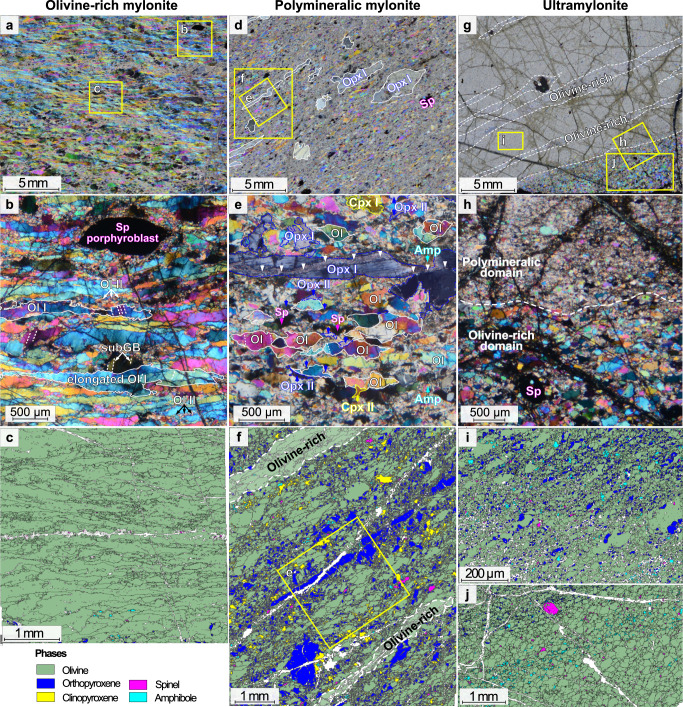
Microstructures of deformed peridotites sampled on the Atobá Ridge. Microphotographs in cross-polarized light (**a**, **b**, **d**, **e**, **g**, **h**) and EBSD phase maps (**c**, **f**, **i**, **j**) illustrating the microstructures of deformed peridotites sampled on the Atobá Ridge. Mylonitic samples present an alternation of olivine-rich (**a**–**c**) and polymineralic domains (**d**–**f**). **a**–**c** Olivine-rich domains contain less than 5% of the other phases (sample COL-DR03-12; Supplementary Table S2). These domains, up to 3 cm thick, are formed by ribbon-shaped olivine porphyroclasts (Ol I; two olivine grains are outlined by white contours) showing undulose extinction and subgrain boundaries (subGB; white dashed lines), and bounded by small, recrystallized olivine grains (Ol II). Rare spinel (Sp) porphyroblasts are also observed. **d**, **e** Orthopyroxene porphyroclasts (Opx I) in polymineralic domains are either strongly elongated or subequant with embayments filled by olivine (Ol) (sample COL-DR03-05). They are occasionally fractured (white arrows in **e**). The fine-grained matrix is composed of olivine (Ol II) + orthopyroxene (Opx II) ± clinopyroxene (Cpx II) + spinel (Sp) ± amphibole (Amp). **g** Ultramylonitic sample also shows alternation of olivine-rich domains (dashed white lines) with rare olivine porphyroclasts and spinel porphyroblasts, and polymineralic finer-grained domains (**j**) (sample COL-DR08-13). **h**, **j** Locally, olivine-rich domains have coarser grain sizes and mark an abrupt transition with fine-grained polymineralic domains. **i** Polymineralic domains are composed of olivine (green) + orthopyroxene (blue) + spinel (pink) + amphibole (cyan) ± clinopyroxene (yellow). **j** EBSD phase map of the transition from olivine-rich, coarse-grained domain to finer-grained polymineralic domain.

Hereafter, we characterize the microfabric of fresh mylonites (serpentinization <5%, Fig. 2A–F) and ultramylonites (Fig. 2G–J) using high-resolution Electron Backscatter Diffraction (EBSD) maps (Supplementary Table S2). The mineralogical composition of these rocks reveals a composition similar to mantle-derived peridotites with the following average composition: olivine (85%), orthopyroxene (7%) and variable amounts of clinopyroxene (3%), spinel (1%) and secondary amphibole ranging from pargasite to (Mg-rich) hornblende (4%; Supplementary Tables S2 and S3). Polymineralic domains in ultramylonites contain far more orthopyroxene (11%) and amphibole (8%) than similar domains in mylonites (Supplementary Table S2). The mineralogical assemblage and the presence of minor amounts of high-temperature amphiboles allow defining these rocks as deriving from a nearly anhydrous protolith with limited retrograde metamorphic equilibration.

**Fig. 3:**
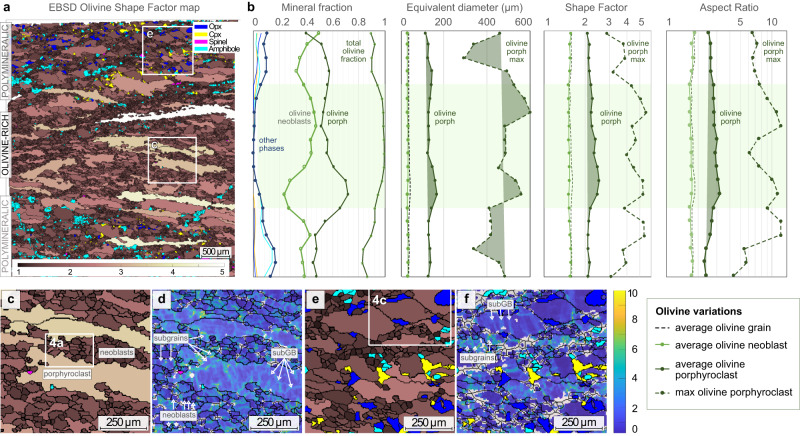
Variations of grain size and shape in a representative mylonite (sample COL-DR03-07). **a** EBSD map of the olivine shape factor, i.e., the ratio between the perimeter of a grain and a circle of equal-area. Olivine shape factor is color coded by different shades of brown: from dark brown = 1 (subrounded grains) to light brown = 5 (elongated grains). The other phases are color coded as in Fig. 2. **b** Integrated vertical variation of mineral abundances, olivine equivalent grain size, olivine shape factor and olivine aspect ratio in map (**a**). Each point corresponds to the averaged parameter value over a 500 µm thick box for the total length of map (**a**). The vertical displacement is 250 µm. The light green band indicates the olivine-rich domain. **c** Detail of the olivine-rich domain: olivine porphyroclasts display elongated shapes (shape factor >4) and very irregular grain boundaries. The size of the neoblasts is similar to the size of the bulges in olivine porphyroclasts. The white box shows the location of Fig. 4a. **d** Kernel Average Misorientation (KAM) map of the area (**c**), showing an orthogonal network of subgrain boundaries (subGB; misorientation close to 10°) forming subgrain cells. **e** Detail of the polymineralic domain: olivine grains display cusp-shaped boundaries with small interstitial orthopyroxene (blue), clinopyroxene (yellow) and amphibole (cyan). The white box shows the location of Fig. 4c. **f** KAM map of area (**e**), showing a lower density of subgrain boundaries (subGB). Some grains have the same patchy pattern observed in the olivine-rich domains (**d**), while most grains are almost strain free, with only few parallel subgrain boundaries.

**Fig. 4:**
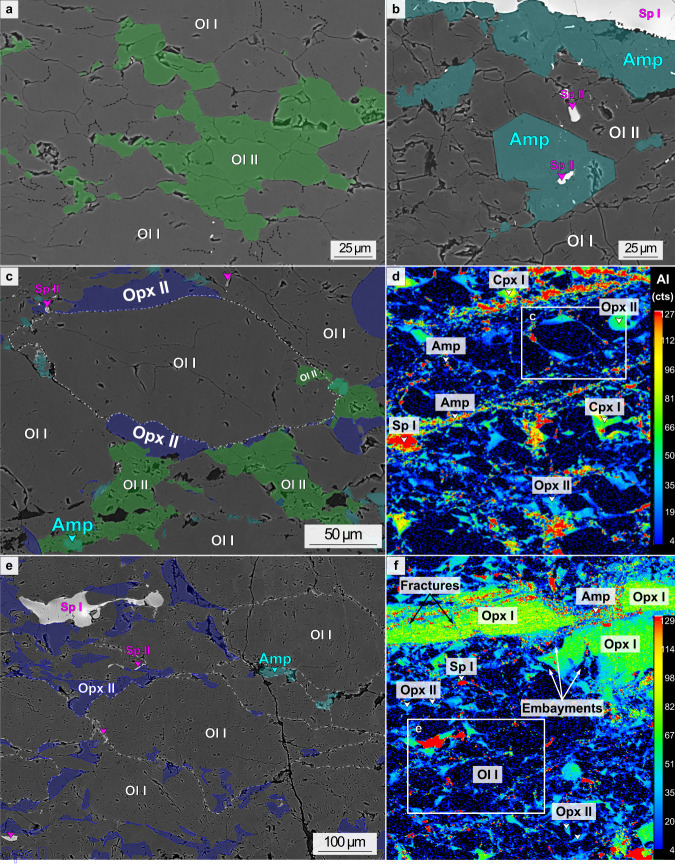
Variation of grain shapes and composition in olivine-rich and polymineralic domains. Backscatter SEM images (**a**–**c**, **e**) and compositional maps of Aluminum (**d**, **f**) showing grain shapes of primary and secondary minerals in mylonite samples. **a** Detail of the olivine-rich domain shown in Fig. 3C, D (sample COL-DR03-07): olivine neoblasts (Ol II, in green) have polygonal shapes with 120° triple junctions. Subgrain boundaries (black dashed lines) form cells with similar sizes. **b** Detail of a transition from olivine-rich to polymineralic domain (sample COL-DR03-12): olivine is bounded by interstitial to polygonal amphibole (Amp, light blue) and interstitial spinel (Sp II). **c** Detail of the polymineralic domain shown in Fig. 3E, F (sample COL-DR03-07): interstitial oriented orthopyroxene (Opx II), spinel (Sp II) and amphibole (Amp) neoblasts form trails along cusp-shaped olivine grain boundaries (Ol I). **d** The compositional map in aluminum (Al) of the same sample highlights the cusp-shaped olivine porphyroclasts (Ol I) and the oriented trails of interstitial pyroxenes and amphibole along olivine grain boundaries. **e** Detail of another polymineralic domain in sample COL-DR03-05. **f** The compositional map in aluminum of the same sample shows the embayments of orthopyroxene porphyroclasts filled by olivine and orthopyroxene (Opx II) neoblasts, but also the decrease in Al content between orthopyroxene porphyroclasts (Opx I) and interstitial neoblasts (Opx II).

Mylonitic samples present interlayered olivine-rich (up to 3 cm thick) and polymineralic domains with porphyroclastic to porphyroblastic textures (Fig. 2B–E). These textures are formed by porphyroclasts embedded in a finer grained matrix of neoblasts, where porphyroclasts are relics of large, plastically deformed olivine (Ol I) and orthopyroxene (Opx I) grains, most of them elongated (Fig. 2B–F). Both porphyroclasts show intracrystalline deformation such as undulose extinction and subgrain boundaries (Fig. 2A, B, D, E). Olivine porphyroclasts grain sizes and shapes correlate to the proportion of mineral phases in the different domains (Fig. 3A–B). Olivine-rich domains have elongated, ribbon-shaped olivine porphyroclasts with irregular grain boundaries (up to 5 mm, average aspect ratio >2.9; Figs. 2A–C and 3A–C; Supplementary Dataset), while polymineralic domains have smaller olivine porphyroclasts with more homogeneous grain shapes (average range of aspect ratio: 1.9–2.3, Figs. 2E and 3A, B) and smooth cusp-shaped boundaries (Figs. 2E and 3E). By contrast, average grain sizes and shapes for olivine neoblasts (Ol II) in the matrix are similar in both domains (Fig. 3B and 4A–C). The rare orthopyroxene porphyroclasts (Opx I) have irregular shapes reaching extreme stretching and elongation (up to 3 mm long; Fig. 2D–F). They show embayments filled by olivine and are occasionally fractured (Figs. 2D–F and 4F).

Internal misorientations also differ in olivine porphyroclasts from the two domains. Olivine porphyroclasts from olivine-rich domains have tightly spaced subgrain boundaries forming orthogonal subgrain cells (Figs. 2B, 3D and 4A), with similar sizes as the bulges in the porphyroclasts grain boundaries and as the olivine neoblasts bounding the porphyroclasts (average range of 30–33 µm; Figs. 2B, 3C, D and 4A; Supplementary Dataset). By contrast, olivine porphyroclasts from polymineralic domains show less internal deformation: they are almost strain free, with only few parallel subgrain boundaries (Figs. 2E and 3F). In addition, small interstitial neoblasts of orthopyroxene and minor phases (spinel ± amphibole ± clinopyroxene) bound olivine grain boundaries and triple junctions (10–59 µm in average range of sizes; Figs. 2E, 3E, F and 4C, E; Supplementary Dataset). These interstitial grains form film-like trails between olivine grains, oriented parallel to the foliation, with highly irregular grain boundaries and cusp-shaped terminations (Figs. 3E, F and 4C–F). Polygonal amphibole crystals are also common, in equilibrium with olivine neoblasts (Fig. 4B, C). Chemically, orthopyroxene interstitial grains (Opx II) have more depleted compositions in Al, Ca compared to orthopyroxene porphyroclasts (Opx I; Fig. 4D, F; Supplementary Table S4).

The most extremely sheared rocks, the ultramylonites, also show interlayering of olivine-rich (>95% olivine) and polymineralic domains (Fig. 2G). The fine-grained matrix (>90%) contains small amphiboles also ranging from pargasite to (Mg-rich) hornblende (Supplementary Table S3) intermixed with the anhydrous phases (olivine + orthopyroxene + spinel ± clinopyroxene) (Fig. 2H, I). Interstitial orthopyroxene and minor phases are aligned along the foliation (Fig. 2I), with similar grain sizes as the surrounding olivine neoblasts (ranging 10–15 µm and 8–23 µm on average for olivine and orthopyroxene, respectively; Supplementary Dataset). Locally, olivine-rich domains have coarser grain sizes with sub-equant, weakly elongated shapes, and rare spinel porphyroblasts (Fig. 2H, J). The transition from the olivine-rich, coarse-grained domain to finer-grained polymineralic domain is well-defined and marked by the abrupt decrease of olivine grain size (Fig. 2G, H).

### Crystal preferred orientation of olivine and orthopyroxene

**Fig. 5:**
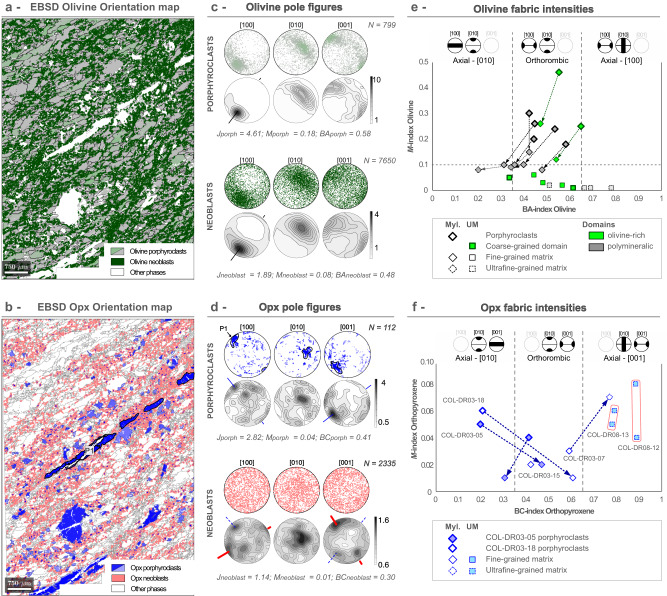
Representative EBSD crystallographic orientations for olivine and orthopyroxene in a mylonite polymineralic domain (sample COL-DR03-05), and fabric intensity evolution plots of the two minerals for the whole data set. **a** EBSD olivine orientation map: porphyroclasts are color coded from light green to gray by increasing the deviation from the mean orientation. Olivine neoblasts are shown in dark green. **b** EBSD orthopyroxene orientation map: orthopyroxene (Opx) porphyroclasts with mean orientation are shown in blue (P1), progressively shading to light blue by increasing misorientation. Orthopyroxene neoblasts are colored in light red. **c**, **d** Pole figures are stereographic projections of the [100], [010], and [001] crystallographic axes of olivine (**c**) and orthopyroxene (**d**) for the EBSD maps (**a**, **b**). All measured pixels in the EBSD map are plotted in the upper panels, with the same color coding as in **a** and **b**. Density contours are plotted in the lower panels (at 1 multiple of a uniform distribution intervals). *N* refers to the number of grains plotted in the pole figures, *J*, *M*, BA, BC to the *J*-, *M*-, BA- and BC-indexes, respectively. **e** Olivine misorientation index *M* versus BA-index (see Supplementary Dataset). The ideal symmetries for axial-[010] (BA < 0.35), orthorhombic (0.35 < BA < 0.65), and [100]-axial domains (BA > 0.65) are reported on top. The upper limit of misorientation measured in orthopyroxene (see inset **f**) is indicated by the horizontal dashed line. **f** Orthopyroxene misorientation index *M* versus BC-index The ideal symmetries for axial-[010] (BC < 0.35), orthorhombic (0.35 < BC < 0.65), and axial-[001] domains (BC > 0.65) are reported on top.

**Fig. 6:**
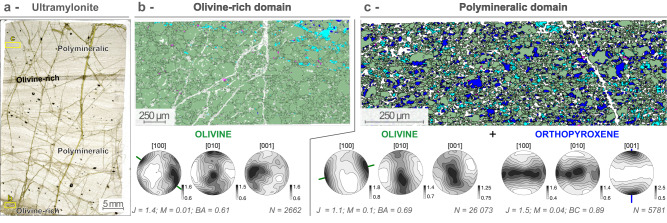
Representative EBSD crystallographic preferred orientations of olivine and orthopyroxene for both olivine-rich and polymineralic domains in an ultramylonite (sample COL-DR08-12C). **a** Microphotograph under natural light of an ultramylonite thin section. Olivine-rich and polymineralic domain can be recognized at the thin section scale. Yellow boxes are locations of details (**b**, **c**). **b**, **c** EBSD maps and corresponding stereographic projections of density contours for the [100], [010], and [001] crystallographic axes of olivine and orthopyroxene (at 1 multiple of a uniform distribution intervals). In olivine-rich domain (**b**), the orientation is orthorhombic (see also Fig. 5E), while in polymineralic domain (**c**) olivine CPO shows an axial-[100] symmetry. Orthopyroxene grains have an axial-[001] symmetry. *N* refers to the number of grains plotted in the pole figures, *J*, *M*, BA, BC to the *J*-, *M*-, BA- and BC-indexes, respectively.

Mineral textures in mylonites and ultramylonites have been investigated by EBSD. The Crystallographic Preferred Orientation (CPO) of olivine and orthopyroxene are presented by orientation maps, stereographic projections and density contours of the distribution (Figs. 5A–D and 6; Supplementary Fig. S1). The strength of each CPO was quantified using the *J* textural and *M* misorientation indexes (Figs. 5E, F and 6B, C; Supplementary Figs. S1-S2 and Supplementary Dataset). Both indexes scale the degree of mineral orientation from random (*J* = 1; *M* = 0) to the CPO of a single crystal (*J* = ∞; *M* = 1; see Methods for further details).

Crystallographic axes distribution in mylonites show that all olivine grains from both domains have [100] maxima close to the shear direction (or lineation) and weaker point concentrations or girdles on [010] and [001] axes at high angle to the shear direction (Fig. 5C, Supplementary Fig. S1). These patterns suggest activation of the [100]{0kl} slip system. Elongation of olivine porphyroclasts parallel to the [100]_Ol_ axis underline the lineation (Fig. 5A). Olivine neoblasts from both domains have similar, but more dispersed CPO pattern than olivine porphyroclasts (*J* < 3 in polymineralic domains vs *J* > 4.61 for olivine porphyroclasts; Fig. 5C, Supplementary Fig. S1 and Supplementary Dataset). The rare orthopyroxene porphyroclasts have moderate CPOs (*J* > 3) correlated to olivine CPOs, with [001]_Opx_ axis subparallel to the lineation and to [100]_Ol_, and [100]_Opx_ at high-angle to [100]_Ol_ (Fig. 5D). This slip system is confirmed by the elongation of orthopyroxene porphyroclasts along [001]_Opx_, which is only possible to grains oriented for glide dislocation on the (100)[001]^32^ (Fig. 5B, D). In all samples, orthopyroxene neoblasts display a different, and more dispersed CPO pattern than orthopyroxene porphyroclasts (*J* < 3), characterized by [001]_Opx_ maxima perpendicular to the lineation and to [100]_Ol_ axis, and [100]_Opx_ axis at small angle to [100]_Ol_ axis (Fig. 5D, Supplementary Fig. S1). In all studied polymineralic domains, including ultramylonitic samples, olivine and orthopyroxene neoblasts have [001]_Opx_ perpendicular to [100]_Ol_ and subparallel to [001]_Ol_ (Figs. 5C, D and 6C, Supplementary Fig. S1).

To fully characterize the CPO of olivine and orthopyroxene, we used the eigenvalues to calculate the BA- and BC-indices for olivine and orthopyroxene respectively (see Methods). The BA-index quantifies the CPO symmetry according to [100] and [010] intensities, which is relevant for olivine as its preferred direction is [100] (Fig. 5C and 6B, C). This index scales from 0 to 1, with BA = 0 for a perfect axial-[010] symmetry, BA = 0.5 for an orthorhombic symmetry and BA = 1 for axial-[100] symmetry (Fig. 5E). The BC-index is calculated in a similar way, using intensities on [010] and [001] axes instead, which are more appropriate for orthopyroxene having the maximum pole concentrations on [001]^31^ (Figs. 5D and 6C).

In mylonites, olivine porphyroclasts have BA values corresponding to orthorhombic symmetries (0.42–0.65; Fig. 5E), matching the pole figures (Fig. 5C, Supplementary Fig. S1). The strength of porphyroclasts fabric varies from high in olivine-rich domains (*M* values: 0.25–0.46) to moderate in polymineralic domains (*M* values: 0.18–0.3; Fig. 5E, Supplementary Figs. S1 and S2). It further decreases in neoblasts, resulting in weaker fabrics with lower *M* (<0.26) and BA values (<0.54; Fig. 5E, Supplementary Fig. S2). Olivine neoblasts in olivine-rich domains inherit the orthorhombic symmetry of the porphyroclasts (BA-indices of 0.47–0.54; Fig. 5E), while those from polymineralic domains slightly shift towards [010]-symmetries (BA-indices of 0.31–0.48), with weaker fabrics (*M* < 0.15; Fig. 5E, Supplementary Fig. S2). Orthopyroxene CPOs are overall weaker than olivine ones (*M* < 0.1, Fig. 5F). Symmetries shift from mostly axial-[010] in porphyroclasts (BC values: 0.20–0.41), to very weak (*M* ≈ 0.03) orthorhombic symmetries in neoblasts (BC values of 0.3–0.77; Fig. 5F).

In ultramylonites, olivine fabric intensities are weaker than in mylonites and progressively randomize (*M* → 0; Figs. 5E and 6B, C). In addition, while olivine from olivine-rich domains share similar orthorhombic CPOs with those from mylonites (BA = 0.34-0.61; Figs. 5E and 6B), olivine from polymineralic domains do not follow the same evolution. The high BA values (>0.51) indicate a shift towards axial-[100] symmetries, with [100] maxima and girdles on [010] and [001] axis (Fig. 5E and 6C). Orthopyroxene CPOs, despite being also weak in ultramylonites (*M* < 0.1), do not completely randomize and are even stronger than olivine CPOs from the same polymineralic domains (*M* ≥ 0.04; Fig. 5F and 6C). BC-values are higher (≥0.78), which are consistent with the axial-[001] symmetries observed in pole figures (Fig. 6C).

### Deformation conditions

Deviatoric stresses can be derived from the well-established correlation between the grain size of synkinematic olivine neoblasts in monomineralic domains and the applied deviatoric stresses^10,33^. Using the piezometer of Van der Wal et al.^33^, the olivine average grain size in olivine-rich domains (30–33 µm in average) gives average yield stress estimates of 87–93 MPa (Table 1 and Supplementary Dataset).Olivine-rich domains from ultramylonites, having similar average grain sizes (28–33 µm), give comparable average stresses of 87–98 MPa (Table 1, Supplementary Dataset). Slightly lower stresses (58–72 MPa) can be inferred for olivine-rich coarser-grained domains in ultramylonites (42–56 µm in average; Table 1, Supplementary Dataset).

**Table 1:** Estimated deviatoric stresses and temperatures for neoblasts and porphyroclasts of olivine and orthopyroxene

Sample			Olivine neoblasts				OPX porphyroclasts						OPX neoblasts		
							Core			Rim					
Name	Texture	Microstructure	*d* (µm)	Mean calculated stress (MPa)	Min. stress (MPa)	Max. stress (MPa)	*T* Ca-in-Opx (°C)	*T* Al-Cr in Opx (°C)	number of points	*T* Ca-in-Opx (°C)	*T* Al-Cr in Opx (°C)	number of points	*T* Ca-in-Opx (°C)	*T* Al-Cr in Opx (°C)	number of points
COL-DR03-05	Mylonite	Polymineralic domain	13 ± 12	–	–	–	–	–	–	859 ± 9	944 ± 11	(3)	772 ± 31	750 ± 45	(3)
COL-DR03-07	Mylonite	Polymineralic domain	29 ± 13	–	–	–	867 ± 19	903 ± 64	(6)	804 ± 1	852 ± 45	(2)	777 ± 20	743 ± 14	(5)
COL-DR03-07	Mylonite	Olivine-rich domain	30 ± 13	93	53	359	–	–	–	–	–	–	–	–	–
COL-DR03-12	Mylonite	Olivine-rich domain	33 ± 20	87	37	190	–	–	–	–	–	–	–	–	–
COL-DR03-15	Mylonite	Polymineralic domain	24 ± 14	–	–	–	–	–	–	–	–	–	797 ± 23	735 ± 51	(8)
COL-DR08-12	Ultramylonite	Olivine-rich coarse-grained domain	42 ± 29	72	22	157	–	–	–	–	–	–	–	–	–
COL-DR08-12	Ultramylonite	Olivine-rich domain	28 ± 17	98	28	359	–	–	–	–	–	–	–	–	–
COL-DR08-13	Ultramylonite	Olivine-rich coarse-grained domain	56 ± 41	58	19	157	–	–	–	–	–	–	–	–	–
COL-DR08-13	Ultramylonite	Olivine-rich domain	33 ± 15	87	37	157	–	–	–	–	–	–	–	–	–
COL-DR08-13	Ultramylonite	Polymineralic domain	10 ± 6	–	–	–	864 ± 4	867 ± 16	(2)	847 ± 2	942 ± 0.1	(2)	768 ± 48	740 ± 62	(4)

Deviatoric stresses are inferred from olivine neoblasts diameter (*d*) in olivine-rich domains using the piezometer of Van der Wal^33^ (Supplementary Dataset). Temperatures are estimated based on Ca-in-Opx^34^ and Al-Cr-in-Opx^35^ thermometers at 0.6 GPa on orthopyroxene porphyroclasts and neoblasts (Supplementary Table S4). Both thermometers have an estimated uncertainty of ±20 °C given by the authors.

Minimum deformation temperatures have been estimated in polymineralic domains using two geothermometers based on orthopyroxene composition: *T*_Ca-in-Opx_^34^ and *T*_Al-Cr-in-Opx_^35^ (see Methods). Both thermometers have an estimated uncertainty of ±20 °C^34,35^. The widespread presence of spinel and the absence of plagioclase in the deformed mineral assemblage suggest that the deformation must have occurred at spinel facies conditions. We then calculate the *T*_Ca-in-Opx_ at nominal pressures of 0.6 GPa (minimum pressure) that represents the spinel to plagioclase boundary transition for depleted lherzolite^36^. Minimum deformation temperatures calculated in orthopyroxene porphyroclasts average 804 °C and 852 °C using *T*_Ca-in-Opx_^33^ and *T*_Al-Cr-in-Opx_^34^, respectively (Table 1 and Supplementary Fig. S4). Orthopyroxene neoblasts in mylonites have slightly lower temperatures averaging 772 °C (*T*_Ca-in-Opx_) and 735 °C (*T*_Al-Cr-in-Opx_) (Table 1, Supplementary Fig. S4), suggesting that these rocks have experienced partial re-equilibration during exhumation. The only ultramylonitic sample analyzed gives average temperatures of 864 °C (*T*_Ca-in-Opx_) and 867 °C (*T*_Al-Cr-in-Opx_) for the orthopyroxene porphyroclasts, and temperatures of 768 °C (*T*_Ca-in-Opx_) and 740 °C (*T*_Al-Cr-in-Opx_) for orthopyroxene neoblasts (Table 1, Supplementary Fig. S4). These temperatures may correspond to the closure temperatures of the Ca-Mg and Al-Cr exchanges between pyroxenes, thus representing minimum deformation temperatures of the system.

## Discussion

Deformed peridotites from the Atobá ridge record strong viscoplastic deformation of primary minerals (olivine, pyroxenes, spinel), resulting in extreme grain size reduction. The deformation mechanisms are discussed below.

Olivine in both mylonites and ultramylonites show a dominant glide along [100] axis with activation of both (010)[100] and (001)[100] slip systems (Fig. 5C and 6B, C, Supplementary Fig. S1). These slip systems, which can be summarized as [100]{0kl} slip system, are commonly observed during high temperature (>1000 °C), low strain deformation^37^ with dry to low water content within olivine crystals^38,39^. In olivine-rich domains, the presence of an orthogonal network of subgrain boundaries in olivine porphyroclasts (Figs. 3D and 4A), the similar sizes of the subgrain cells in porphyroclasts and that of neoblasts bounding the porphyroclasts (Figs. 3D and 4A), the occurrence of 120° triple junctions (Fig. 4A) and the formation of ribbon-shaped porphyroclasts (Figs. 2A–C and 3A) point to dislocation creep as the main deformation mechanism during dynamic recrystallization. This mechanism leads to grain size reduction by subgrain rotation, and the activation of [100]{0kl} as the main slip system^15,16,37^. Olivine-rich domains from both mylonites and ultramylonites have similar grain size distribution, while grain size further decreases in polymineralic domains (Fig. 6C; Supplementary Dataset). This difference is also reflected by the variation of the aspect ratio (Fig. 3B), the decrease of both fabric strength (Fig. 5E, Supplementary Fig. S2) and of the density of misorientations (Fig. 3D, F), suggesting that deformation and grain size reduction were preferentially localized in the polymineralic domains.

In polymineralic domains, olivine has a similar but weaker CPO pattern than in olivine-rich domains (Figs. 5C, E and 6C), suggesting a decreasing contribution of dynamic recrystallization, hence dislocation creep, to grain size reduction, and the activation of diffusion creep mechanisms. The occurrence of (rare) quadruple junctions indicates that grain boundary sliding has been active (Figs. 3E, F and 4B, C)^10,19^. Yet, the cusp-shapes of olivine grains (Ol I; Figs. 3E, F and 4C–F), the lower amount of internal misorientations (Figs. 2E and 3F) and the increasing strength of the orthopyroxene fabric with grain size reduction (Fig. 5F) suggest an increased contribution of diffusive, grainsize sensitive creep, other than grain boundary sliding^19,20^. The polymineralic nature of these domains (Figs. 2E, F and 4) is also known to inhibit grain growth and therefore contribute to diffusion creep^10,17,40^. The presence of orthopyroxene porphyroclasts with sinuous boundaries and micro-embayments filled by olivine (Figs. 2E-F, 4F), the cusp-shaped olivine grains (Figs. 3E, F and 4C, E), along with small oriented orthopyroxene (Opx II), clinopyroxene (Cpx II) and amphibole (Amp) crystals at olivine-olivine grain boundaries and triple junctions (Figs. 2E, 3E, F, and 4), suggest local dissolution and precipitation in presence of fluids, as documented in other subcontinental contexts^16,18–20,41–43^. The increasing proportion of orthopyroxene neoblasts and decreasing of clinopyroxene with grain size reduction are additional evidence for dissolution of pyroxene porphyroclasts followed by interstitial precipitation of orthopyroxene neoblasts and minor hydrous phases (amphibole), following the relation: Opx I + Cpx I + Ol I + fluid → Opx II + Amp + Ol II ± Cpx II. Accordingly, the composition of the orthopyroxene neoblasts shifts to lower Al and Ca contents compared to the porphyroclast composition (Fig. 4D, F; SupplementaryTable S4).

The weak, but non-random CPO of orthopyroxene neoblasts with [001]_Opx_ axes at high angle to the lineation and to [001]_Opx_ porphyroclasts axes (both parallel; Figs. 5D and 6C) show that this CPO is clearly not inherited from orthopyroxene porphyroclasts^19,20^. Instead, orthopyroxene neoblasts have their [100]_Opx_ axes sub-parallel to the lineation and to [100]_Ol_, which also supports that they result from precipitation rather than dynamic recrystallization. This uncommon orthopyroxene CPO has been characterized to occur during deformation assisted by hydrous fluid/melt in grainsize sensitive creep, with dissolution and local precipitation of orthopyroxene in sub-continental contexts^19,41,43^, ophiolites^16,18,20^ and more recently in modern oceanic domains^44^.

**Fig. 7:**
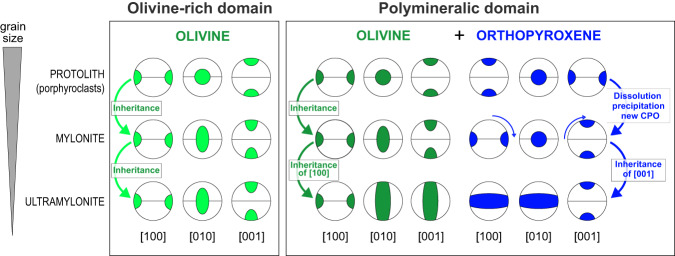
Summary cartoon of Crystallographic Preferred Orientation (CPO) for both olivine and orthopyroxene in peridotites from the Atobá ridge during progressive grain size reduction in olivine-rich (left panel) and polymineralic domains (right panel). The protolith has orthorhombic CPO typical of high temperature conditions: [100](001) for olivine and [001](100) for orthopyroxene (Fig. 5E, F). At intermediate degrees of deformation (mylonite), the CPO of olivine neoblasts, in both olivine-rich and polymineralic domains, is mostly inherited from the protolith porphyroclasts. By converse, orthopyroxene neoblasts in polymineralic domains develop a new CPO, mimicking that of the olivine, with [001]_Opx_ orthogonal to [100]_Ol_ (right panel). At extreme deformation degrees (ultramylonites), both olivine and orthopyroxene develop axial symmetries shown by the girdles but preserve the orthogonal relationship between [001]_Opx_ and [100]_Ol_.

The shift of olivine CPO towards axial-[010] symmetries is also consistent with deformation in presence of low amounts of hydrous fluid/melt^41,43,45^ (Fig. 5E, Supplementary Fig. S2). The activation of dissolution-precipitation creep is possibly guided by the proportion of orthopyroxene and minor phases in the initial mineralogical assemblage of the protolith (Fig. 3B, Supplementary Fig. S2; Supplementary Table S2). Indeed, this process is not observed in olivine-rich domains where olivine neoblasts mostly inherit their fabric from porphyroclasts with dominant [100] glide, and where the grain size reduction is mostly governed by dislocation creep, leading to a similar CPO (Figs. 5E, 6B and 7; Supplementary Figs. S1 and S2).

Polymineralic domains in ultramylonitic samples are characterized by further grain size reduction and olivine and orthopyroxene grains having both axial symmetries with dominant glides on [100] and [001] axes, respectively (Fig. 6C). These axial symmetries are supported by the high BA values for olivine (BA > 0.50, Figs. 5E and 6C) and BC values for orthopyroxene neoblasts (BC > 0.78; Figs. 5F and 6C). The orthogonal relationship between [100]_Ol_ and [001]_Opx_ axes suggests that dissolution-precipitation creep was also active during the formation of ultramylonites (Fig. 6C). We therefore propose that the formation of ultramylonites resulted from further strain micro-localization in polymineralic domains, at high to low temperatures (*T* > 770 °C, Table 1), resulting in axial symmetries for both olivine and orthopyroxene phases (Fig. 7).

In both mylonites and ultramylonites, the synkinematic presence of fluid in the system is attested by the nucleation of high temperature (>800 °C)^46^ amphiboles coexisting with olivine neoblasts (Figs. 2I, J, 3A, E, F, 4B, C and 6C), and compositionally ranging from pargasite to hornblende (Supplementary Table S3). Such temperatures are consistent with those estimated on orthopyroxene using the *T*_Ca-in-Opx_^34^ and *T*_Al-Cr-in-Opx_^35^ thermometers (Table 1 and Supplementary Fig. S4). In addition, the presence of spinel and the absence of plagioclase in St. Paul mylonites and ultramylonites indicate that this ductile deformation stage occurred at spinel facies conditions, i.e. at depths corresponding to pressures >0.6 GPa^36^, which raises questions on the origin of the fluid. Similar high-temperature synkinematic amphiboles have been described in oceanic contexts in deformed peridotite samples from Shaka^5,6,9^ and Vema^11^ OTFs, at magma-starved oceanic detachment faults^44,47–49^ but also in orogenic massifs^19,41,48^. These hydrous minerals can either be supplied from seawater-derived fluids percolating down to the root of transform or detachment faults^5,6,11,44,47,48^, or from limited magma-driven metasomatism of mantle-derived rocks^11,41,47^. The first option is here supported by high-Cl content of amphibole and lack of magma related element enrichment as Ti, Na and K (Supplementary Tables S3 and S4). In addition, recent seismic studies along the St. Paul TS recorded seismicity up to 20 km below the seafloor, attesting for deep lithospheric fracturing to sustain fluid circulation^8^. Consequently, we propose that amphibole (neoblasts) in St. Paul mylonites crystallized after seawater-derived fluids and not from magma-driven metasomatism reported elsewhere in the St. Paul region^25^.

Primary mineral heterogeneities (i.e. olivine-rich vs. polymineralic domains) in the peridotite protolith are enhanced during localization of deformation; they can promote micro-scale rheological contrasts, such as between orthopyroxene and olivine, leading to local stress concentrations and strain focusing, thus promoting local grain size reduction by dislocation-accommodated mechanisms^19,27,31,44^. This process, in turn favors fluid-assisted reactions and formation of an interconnected network^45^, leading to olivine weakening, modification of the olivine dominant slip system and mantle viscosity reduction^7,38,41,45,50^. The introduction of small quantities of hydrous fluid/melt at depths would also enhance creep rates^50^, favouring grain size reduction and progressive transition to grain size sensitive creep, and further strain localization^7,22,41,47,48^. Fluids are therefore key to localize strain at depths and can potentially induce a major rheological weakening of the lithospheric mantle^38,39,50^. We hence propose that the diffuse grain size reduction observed in St. Paul mylonites results from a combination of deformation mechanisms depending on the initial mineralogy. Dislocation creep is dominant in olivine-rich domains, while fluid-assisted dissolution-precipitation creep is the main mechanism in polymineralic domains (Fig. 7). These deformation mechanisms document the weakening processes acting at depth along an oceanic transform fault.

The petrographic microstructural characters described here, i.e. the extensive grain size reduction, the interlayered olivine-rich and polymineralic domains, the occurrence of syn-deformational high-temperature hydrous phases^5,6,9–12,51^, and more rarely the orthopyroxene CPO mimicking that of the olivine^51^, have been reported in other transform domains along the MOR system. However, all these observations have not previously been interpreted as pertaining to fluid-assisted dissolution-precipitation creep, which could represent the leading mechanism during localization of deformation at OTFs. Yet, recent petrological studies of various OTFs mylonites, such as those from the Shaka and Garret Fracture Zones^5–7,12^, support this assumption by showing positive feedback loop in the brittle-ductile transition zone between seismic-driven fluid infiltration, weakening, grain size reduction, shear and fluids.

The importance of high temperature fluid-assisted dissolution-precipitation creep as an effective deformation process in the upper mantle is already known for subcontinental crust^41^, oceanic crust metamorphosed at HT conditions^52,53^, mantle and ophiolites^16,19,20,43^. Recently, oceanic detachment faults in magma-starved contexts also evidenced limited fluid-assisted deformation at the root zone of the detachment^44,47–49,54^. We therefore suggest that fluid-assisted dissolution-precipitation creep could be a major rheological process controlling strain localization and faulting at plate boundaries.

## Methods

Foliation and lineation of selected samples are based on the shape preferred orientation (SPO) of pyroxene and spinel grains; thin sections were cut perpendicular to the foliation and parallel to lineation, when visible (XZ plane). All thin sections were polished using a Vibromet Buehler with a colloidal silica solution during 1H15 to improve the surface of the thin sections for an optimal microstructural and microprobe analysis.

### SEM data acquisition

Scanning electron microscopy (SEM) imaging and analysis were done at 20 kV with a FEI Quanta 200 SEM coupled with an OXFORD X-MAXN Silicon Drift Detector (detector size: 80 mm^2^) at IFREMER (Plouzané, France).

### CPO data acquisition

Crystallographic preferred orientation (CPO) of olivine, pyroxenes, spinel, and amphibole was measured in 8 selected samples using a JEOL JSM-7100F TTLS LV at the Centre de Microcaractérisation Raimond Castaing located in Toulouse, France. EBSD maps were recorded with 20 kV acceleration voltage and 15 mm working distance. For each thin section, high-resolution CPO maps were obtained for polymineralic and olivine-rich domains, with a grid step from 0.2 to 8 μm, depending on the observed minimum grain size (Supplementary Table S2). Among them, details of the matrix were obtained with a grid step of 0.2–2 μm. Post-acquisition treatment consisted in deleting wild spikes and filling non-indexed pixels with ≥7-8 neighbors with orientations coherent to their average orientation.

CPO data and modal proportions were processed using the Channel 5.4 software and MTEX MATLAB toolbox (http://mtex-toolbox.github.io/)^55,56^. The orientation distribution functions (ODFs) were calculated using a “de la Vallée Poussin” kernel function with a half-width of 15°. All CPOs are presented as one crystallographic orientation per grain in pole figures (lower hemispheric stereographic projections, Figs. 5C, D and 6; Supplementary Fig. S1). To analyze quantitatively the microstructure, we used the grain recognition routine in MTEX^55^ with a misorientation threshold of 15° between two neighboring pixels for defining grain boundaries. Subgrain boundaries were identified by misorientations between neighboring pixels of 2–10°. Intragranular deformation was characterized by analysis of the Kernel Average Misorientation (KAM) and Misorientation relative to the Mean Orientation of the grain (Mis2Mean) maps. Except for one sample (COL-DR01-18), small grains are well defined, and their grain size and shape distributions were quantified using MTEX (Fig. 3B; Supplementary Dataset). The average grain size corresponds to the mean equivalent diameter calculated using the grain ellipsoid fit, and the Feret diameter quantifies the maximum elongation. Average olivine grain sizes in olivine-rich domains were multiplied by 1.2 to correct for sectioning effects then used to estimate paleostresses based on the olivine paleopiezometric relation of Van der Wal et al.^33^.

MTEX was also used to calculate fabric strengths using the *M*-^57^ and *J*-indices^58^, and to calculate the BA-index^59^. The *J*-index is the volume-averaged integral of the squared orientation densities^58^, and has been derived from ODFs. The *J*-index ranges from 1 for a random fabric to infinity for a single crystal, while the *M*-index ranges from 0 for a random fabric to 1 for a single crystal^60^. The latter is based on the distribution of uncorrelated misorientation angles, which are the angles of rotation around a common axis used to bring two crystal lattices on the same orientation^57^. The BA-index is the fabric symmetries indices and is calculated as 0.5*(2−(P010/(G010 + P010))−(G100/(G100 + P100)))^59^. In the case of olivine, the values varies between 0 and 1, where BA values < 0.35 represent an axial-[010] fabric, BA values between 0.35 and 0.65 an orthorhombic fabric and BA > 0.65 corresponds to an [100]-axial fabric.

### Microprobe data

In situ major element concentrations of pyroxenes and amphiboles were measured using a JEOL JXA-8200 electron microprobe at Dipartimento di Scienze della Terra, Università degli Studi di Milano (Italy). The accelerating voltage was fixed at 15 kV and beam current at 15 nA. The spot size was 1 μm. Counting time was 30 s on the peak and 10 s on the backgrounds. A set of natural standards was used for calibration. Amphibole and pyroxenes compositions are reported in Supplementary Tables S3 and S4, respectively. Temperatures of deformation are estimated from orthopyroxene composition (Supplementary Table S4), using two geothermometers based on Ca-Mg and Al-Cr exchanges in orthopyroxene: *T*_Ca-in-Opx_^34^ and *T*_Al-Cr-in-Opx_^35^. Both thermometers have an estimated uncertainty of ±20 °C given by the authors. For equilibrium temperatures estimated with *T*_Ca-in-Opx_, we chose the pressure of 0.6 GPa, which is the minimum pressure at which the spinel to plagioclase transition occurs^36^. As the recrystallized assemblage does not contain any plagioclase, the deformation must have occurred at depths corresponding to pressures >0.6 GPa. The equilibrium temperatures calculated with the above geothermometers therefore correspond to the minimum deformation at which grain size reduction occurs in St. Paul mylonites and ultramylonites.

Composition maps (Fig. 4D, F) were acquired with 3–5 µm spatial resolution using a Cameca SX 100 electron microprobe at the Centre Microsonde Ouest located at Plouzané (France). We used the following analytical conditions: accelerating voltage at 15 kV, beam current at 15 nA, dwell time of 100 ms.

## Supplementary information

Supplementary Information

Description of Additional Supplementary Files

Supplementary Dataset

## Data Availability

Supplementary Information is available for this study: dredges coordinates are provided in Supplementary Table S1, mineralogical proportions and EBSD step sizes of the maps used in this study in Supplementary Table S2. Amphibole and orthopyroxene compositions are shown in Supplementary Tables S3 and S4, respectively. Measured grain sizes and shapes for olivine and orthopyroxene are provided as Supplementary Dataset. EBSD and compositional data used for the maps in this study are available in Figshare: 10.6084/m9.figshare.22561336.
