# Supplementary material for: Fluid-assisted grain size reduction leads to strain localization in oceanic transform faults
Source: Nat Commun. 2023 Jul 10;14:4087. doi: 10.1038/s41467-023-39556-5 (PMC10333221; doi:10.1038/s41467-023-39556-5)
Supplement: Supplementary file 1 — Supplementary Information [file 41467_2023_39556_MOESM1_ESM.pdf]

Supplementary Information

Supplementary Information is available for this study: dredges coordinates are provided in Table S1, mineralogical proportions and EBSD step sizes of the maps used in this study in Table S2. Amphibole and orthopyroxene compositions are shown in Tables S3 and S4, respectively. Measured grain sizes and shapes, and calculated EBSD indexes (*J-, M-,* BA-indexes), for neoblasts and porphyroclasts of olivine and orthopyroxene are provided as Supplementary Dataset. EBSD and compositional data used for the maps in this study are available in Figshare: https://doi.org/10.6084/m9.figshare.22561336.

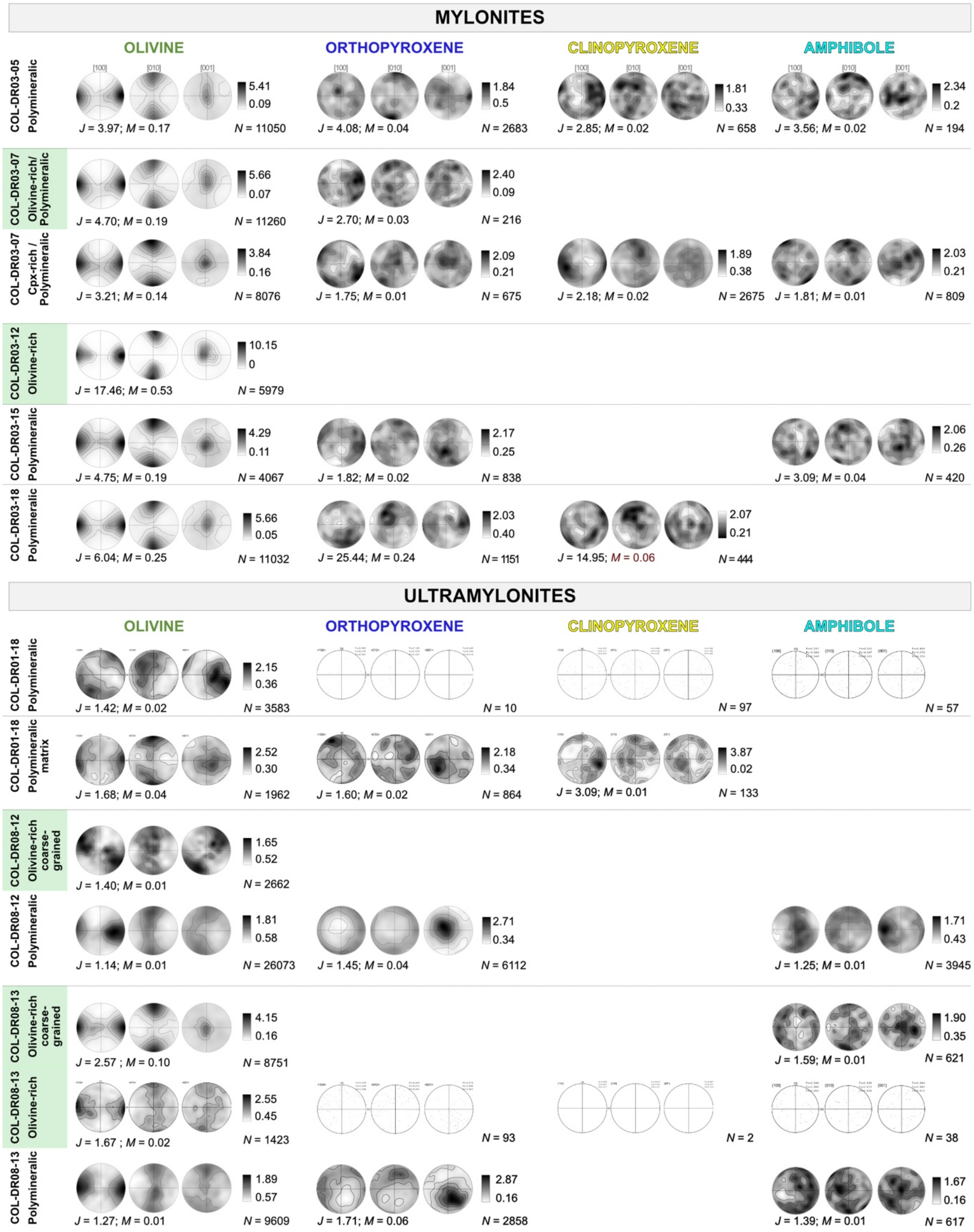

Supplementary Figure S1: **Poles figures are stereographic projections of the [100], [010], and [001] crystallographic axes of olivine, orthopyroxene, clinopyroxene and amphibole plotted in density contours (at 1 multiple of a uniform distribution intervals)**. All pole figures

are oriented with the maximum concentration of olivine [100] and [010] axes are parallel to the E–W and N–S directions of the pole figures, respectively. When possible, CPO for both olivine-rich and polymineralic domains are shown. *N* refers to the number of grains plotted in the pole figures, *J* and *M* to the *J*- and *M*-indexes, respectively (see Supplementary Dataset). Pole figures are shown in stereographic projections when $N < 100$.

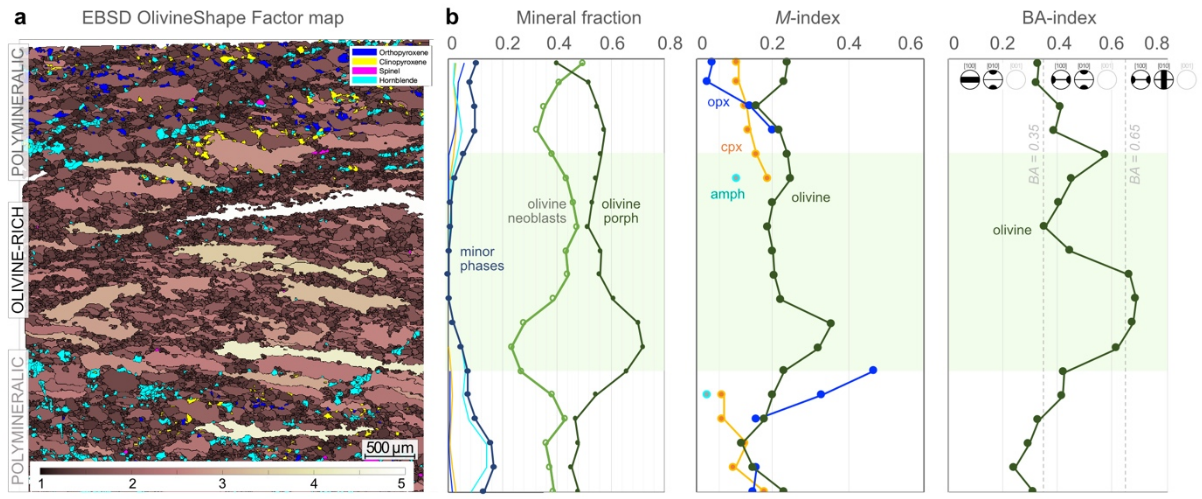

Supplementary Figure S2: **Variation of grain size and fabric intensity in a representative mylonite (sample COL-DR03-07, see Fig. 3).** (a) EBSD map of the olivine shape factor, i.e. the ratio between the perimeter of a grain and a circle of equal-area. Olivine shape factor is color coded by different shades of brown: from dark brown = 1 (subrounded grains) to light brown = 5 (elongated grains). The other phases are color coded as in Fig. 2. (b) Integrated vertical variation of mineral abundances, olivine general *M*- and BA-indexes in map (a) (see Supplementary Dataset). Each point corresponds to the averaged parameter value over a 500 µm thick box for the total length of map (a). The vertical displacement is 250 µm. The light green band indicates the olivine-rich domain. Olivine fabric intensity increases with the fraction of porphyroclasts and the decrease of minor phases.

Supplementary Table S1: **Depths and positions of the dredges done on the Atobá ridge during COLMEIA cruise (Maia, 2013; R/V L'Atalante, https://doi.org/10.17600/13010010).** Positions are based on on-bottom/off-bottom positions and recalculated from ship positions. Abbreviations: longitude (long.), and latitude (lat.), Depth below sea level (b.s.l.).

	Dredge coordinates					Proportions of rocks recovered by dredging (%)					
Dredge	Lat. N, On Bottom	Long. W, On Bottom	Lat. N, Off Bottom	Long. W, Off Bottom	Depth b.s.l (m)	Peridotite	Gabbro	Dolerite	Basalt	Carbonate	Mylonite
DR1	0.8238	-29.6589	0.8508	-29.6757	-2040	-	-	-	-	5	95
DR3	0.9172	-29.2239	0.9355	-29.2272	-1160	-	-	-	-	-	100
DR8	0.9522	-29.0442	0.9748	-29.0441	-3535	-	-	-	-	-	100

Supplementary Table S2: **Deformation, microstructural domain, mineral proportions and EBSD step size (in µm) of the different maps acquired through EBSD method in mylonites and ultramylonites from the Atobá ridge**. Maps marked with an asterisk (*) contain olivine-rich and polymineralic domains. Consequently, proportions have been calculated for each domain separately. Abbreviations: Orthopyroxene (Opx) – Clinopyroxene (Cpx).

				Modal percentages (of mapped areas)						
**MAPS**	**Sample name**	**Deformation**	**Microstructure**	**Olivine**	**Opx**	**Cpx**	**Amphibole**	**Spinel**	**Opx + Minor phases**	**EBSD Step size (µm)**
map1	COL-DR03-05	Mylonite	Polymineralic	79%	17%	4%	1%	0%	22%	8
map2	COL-DR03-05	Mylonite	Polymineralic	88%	8%	2%	0%	1%	11%	8
map3	COL-DR03-05	Mylonite	Polymineralic matrix	91%	5%	0%	4%	0%	9%	1
mapC1	COL-DR03-07	Mylonite	Olivine-rich*	99%	0%	0%	1%	0%	1%	5
mapC1	COL-DR03-07	Mylonite	Polymineralic*	89%	2%	2%	7%	0%	11%	5
mapA2	COL-DR03-07	Mylonite	Polymineralic/Cpx-rich domain	70%	4%	23%	2%	0%	29%	4
mapA3	COL-DR03-07	Mylonite	Polymineralic matrix	78%	8%	8%	6%	0%	22%	1
mapC4	COL-DR03-07	Mylonite	Polymineralic matrix	81%	8%	7%	4%	0%	19%	1
map1	COL-DR03-12	Mylonite	Olivine-rich	100%	0%	0%	0%	0%	0%	4
map2	COL-DR03-15	Mylonite	Polymineralic	90%	7%	0%	2%	0%	9%	3
map1	COL-DR03-18	Mylonite	Polymineralic	85%	12%	3%	0%	0%	15%	7
map2	COL-DR01-18	Ultramylonite	Polymineralic	83%	0%	4%	2%	10%	16%	8
map1	COL-DR01-18	Ultramylonite	Polymineralic matrix	69%	20%	4%	3%	3%	30%	0.2
mapC3	COL-DR08-12	Ultramylonite	Olivine-rich coarse-grained	98%	0%	0%	2%	0%	2%	5
mapA1	COL-DR08-12	Ultramylonite	Polymineralic	74%	5%	0%	18%	4%	27%	5
mapA2	COL-DR08-12	Ultramylonite	Polymineralic matrix	67%	16%	1%	16%	1%	34%	1
mapC1	COL-DR08-12	Ultramylonite	Polymineralic matrix	79%	14%	0%	7%	0%	21%	2
map1	COL-DR08-13	Ultramylonite	Olivine-rich coarse-grained*	95%	0%	0%	3%	1%	5%	5
map2	COL-DR08-13	Ultramylonite	Olivine-rich	96%	3%	0%	1%	0%	4%	5
map3	COL-DR08-13	Ultramylonite	Polymineralic matrix	85%	12%	0%	1%	0%	13%	0.8

Supplementary Table S3: **Average amphibole composition in St. Paul mylonites and ultramylonites.** Abbreviation: amphibole (amph).

Thin section	Position	#analyses	SiO2	SiO2std	TiO2	TiO2std	Al2O3	Al2O3std	FeO	FeOstd	MgO	MgOstd	MnO	MnOstd
COL-DR03-05	AMPH1	2	45.31	0.40	0.66	0.01	12.78	0.44	2.91	0.01	18.96	0.11	0.05	0.01
	AMPH2	1	43.70		1.23		14.10		3.01		17.94		0.04	
	AMPH3	1	41.44		1.21		14.66		3.25		17.20		0.02	
COL-DR03-7A	AMPH1	2	42.79	0.15	1.53	0.07	14.52	0.02	4.69	0.04	17.57	0.02	0.06	0.01
	AMPH2	2	43.35	0.20	2.32	0.07	14.78	0.09	4.28	0.00	17.07	0.16	0.06	0.02
	AMPH3	2	49.43	0.25	0.40	0.03	8.27	0.54	3.48	0.10	20.21	0.44	0.06	0.02
	AMPH4	1	52.15		0.14		5.10		2.75		21.06		0.07	
COL-DR03-15	Amph in vein	6	48.63	1.11	0.29	0.07	9.21	0.89	3.17	0.20	19.81	0.50	0.04	0.03
	Amph in vein - Rim	1	54.51		0.04		3.56		2.48		22.54		0.00	
COL-DR03-15	AMPH1	2	48.25	0.22	0.32	0.03	9.84	0.26	3.56	0.17	19.61	0.04	0.04	0.00
	AMPH1	4	54.92	1.22	0.14	0.05	3.23	1.24	3.05	0.32	21.88	0.56	0.07	0.01
	AMPH2	2	47.75	0.01	0.36	0.01	10.16	0.15	3.53	0.01	19.38	0.14	0.02	0.01
	AMPH3	1	45.01		0.91		13.41		4.21		17.40		0.00	
	AMPH4	1	43.14		1.12		15.17		4.43		16.69		0.08	
	AMPH5	1	48.19		0.55		9.76		3.45		19.37		0.05	
	AMPH6	1	43.67		1.26		13.65		4.47		17.71		0.12	
	AMPH7	1	48.39		0.34		8.96		3.57		19.71		0.06	
COL-DR08-12A	AMPH1 - vein	2	44.55	0.21	0.57	0.22	12.78	0.06	4.19	0.03	18.00	0.27	0.08	0.02
	AMPH2	2	42.65	0.05	4.50	0.02	13.14	0.00	3.74	0.12	16.48	0.03	0.01	0.01
	AMPH3	1	47.56		0.16		9.89		4.25		19.50		0.03	
COL-DR08-13	AMPH1	1	45.87		0.21		12.13		4.12		18.62		0.04	
	AMPH2	1	48.85		0.18		8.82		3.51		19.83		0.09	
	AMPH3	1	57.17		0.08		0.83		1.91		23.07		0.05	
	AMPH3 RIM	1	46.21		0.74		11.46		3.69		18.52		0.07	
	AMPH4	1	47.08		0.26		9.92		3.54		19.51		0.08	
	AMPH4 RIM	1	52.50		0.12		5.08		2.74		21.55		0.07	
	AMPH5	1	43.00		2.42		13.55		4.03		17.39		0.08	
	AMPH6	1	46.05		0.24		11.57		3.87		18.85		0.04	
	AMPH7	1	43.23		2.28		13.72		4.20		17.65		0.00	

Thin section	Position	#analyses	CaO	CaOstd	Na2O	Na2Ostd	K2O	K2Ostd	Cl	Clstd	TOTAL	Totalstd
COL-DR03-05	AMPH1	2	12.55	0.04	2.81	0.16	0.08	0.01	0.00	0.00	97.16	0.11
	AMPH2	1	12.07		3.02		0.17		0.01		96.83	
	AMPH3	1	12.03		3.21		0.18		0.00		94.88	
COL-DR03-7A	AMPH1	2	11.99	0.07	2.72	0.02	0.08	0.01	0.01	0.00	97.16	0.05
	AMPH2	2	11.91	0.07	2.51	0.00	0.20	0.00	0.01	0.01	97.87	0.43
	AMPH3	2	12.53	0.13	1.48	0.06	0.13	0.02	0.23	0.07	97.04	0.14
	AMPH4	1	13.91		1.02		0.07		0.11		96.94	
COL-DR03-15	Amph in vein	6	12.73	0.08	1.81	0.22	0.15	0.02	0.33	0.03	97.46	0.22
	Amph in vein - Rim	1	12.75		0.61		0.04		0.09		96.82	
COL-DR03-15	AMPH1	2	12.58	0.08	1.83	0.04	0.12	0.04	0.24	0.09	97.76	0.28
	AMPH1	4	12.77	0.09	0.60	0.25	0.05	0.02	0.09	0.01	97.40	0.23
	AMPH2	2	12.61	0.03	1.92	0.00	0.16	0.00	0.27	0.02	97.25	0.07
	AMPH3	1	12.52		2.12		0.19		0.58		97.60	
	AMPH4	1	12.44		2.44		0.25		0.90		97.92	
	AMPH5	1	12.72		1.78		0.15		0.31		97.06	
	AMPH6	1	12.58		2.46		0.19		0.32		97.19	
	AMPH7	1	12.47		1.84		0.12		0.25		96.65	
COL-DR08-12A	AMPH1 - vein	2	12.48	0.04	2.50	0.11	0.35	0.10	0.43	0.05	97.12	0.05
	AMPH2	2	11.95	0.07	2.64	0.04	1.06	0.01	0.03	0.00	97.37	0.17
	AMPH3	1	12.02		2.11		0.13		0.29		96.86	
COL-DR08-13	AMPH1	1	12.55		2.37		0.19		0.29		97.51	
	AMPH2	1	12.64		1.77		0.13		0.20		96.71	
	AMPH3	1	12.98		0.12		0.00		0.03		96.42	
	AMPH3 RIM	1	12.52		2.30		0.17		0.25		96.81	
	AMPH4	1	12.55		2.06		0.15		0.17		96.47	
	AMPH4 RIM	1	12.94		0.94		0.08		0.11		96.29	
	AMPH5	1	11.94		2.63		0.17		0.03		96.66	
	AMPH6	1	12.74		2.15		0.20		0.26		96.78	
	AMPH7	1	12.10		2.51		0.19		0.07		97.18	

Supplementary Table S4: **Average orthopyroxene composition and temperatures estimated with the following thermometers: $T_{\text{Ca-in-Opx}}$[33] for 0.6 GPa and $T_{\text{Al-Cr-in-Opx}}$[34].** Both thermometers have an estimated uncertainty of ± 20°C given by the authors (see Methods).

Thin section	COL-DR03-05				COL-DR03-07						COL-DR03-15		COL-DR08-13					
Texture	Mylonite				Mylonite						Mylonite		Ultramylonite					
Position	Rim		Neoblast		Core		Rim		Neoblast		Neoblast		Core		Rim		Neoblast	
number of analyses	3	std	3	std	6	std	2	std	5	std	8	std	2	std	2	std	4	std
SiO2	56.06	0.10	56.73	0.24	55.03	0.23	56.03	0.76	56.16	0.24	56.45	0.47	55.93	0.16	56.13	0.06	57.15	0.19
TiO2	0.08	0.02	0.01	0.01	0.07	0.03	0.04	0.01	0.04	0.02	0.06	0.03	0.05	0.02	0.01	0.01	0.05	0.02
Al2O3	2.61	0.08	1.41	0.33	3.51	0.40	2.19	0.43	1.88	0.26	1.72	0.56	2.57	0.01	2.35	0.01	1.17	0.39
Cr2O3	0.58	0.02	0.21	0.08	0.37	0.14	0.38	0.08	0.14	0.03	0.13	0.07	0.35	0.01	0.57	0.01	0.13	0.11
FeO	5.72	0.29	6.19	0.07	7.61	0.48	6.78	0.23	7.37	0.35	6.69	0.22	5.81	0.04	5.86	0.12	6.49	0.19
MnO	0.15	0.01	0.16	0.01	0.18	0.04	0.18	0.03	0.19	0.02	0.15	0.03	0.16	0.03	0.11	0.01	0.15	0.03
MgO	34.16	0.09	34.63	0.23	32.44	0.26	33.67	0.55	33.62	0.34	34.11	0.30	33.87	0.34	33.98	0.01	34.17	0.21
CaO	0.51	0.02	0.32	0.06	0.53	0.05	0.38	0.00	0.32	0.04	0.36	0.05	0.52	0.01	0.48	0.00	0.31	0.09
Na2O	0.00	0.01	0.01	0.01	0.03	0.03	0.02	0.00	0.00	0.00	0.01	0.01	0.02	0.01	0.03	0.01	0.02	0.01
NiO	0.05	0.04	0.09	0.01	0.08	0.03	0.06	0.04	0.07	0.04	0.07	0.05	0.07	0.03	0.11	0.02	0.10	0.01
TOTAL	99.92	0.23	99.77	0.11	99.84	0.38	99.75	0.58	99.80	0.44	99.78	0.25	99.35	0.44	99.64	0.09	99.76	0.28
T Ca-in-Opx (°C)	859	9	772	31	867	19	804	1	777	20	797	23	864	4	847	2	768	48
T Al-Cr-in-Opx (°C)	944	11	750	45	903	64	852	45	743	14	735	51	867	16	942	0.13	740	62
