# Supplementary material for: Fluid-assisted grain size reduction leads to strain localization in oceanic transform faults
Source: Nat Commun. 2023 Jul 10;14:4087. doi: 10.1038/s41467-023-39556-5 (PMC10333221; doi:10.1038/s41467-023-39556-5)
Supplement: Supplementary file 2 — Description of Additional Supplementary Files [file 41467_2023_39556_MOESM2_ESM.pdf]

<u>**Description of Additional Supplementary Files**</u>

Supplementary Dataset: Grain sizes and shapes, and calculated EBSD indexes (*J-, M-,* BA-indexes) for neoblasts and porphyroclasts of olivine and orthopyroxene. Maps marked with an asterisk (*) contain olivine-rich and polymineralic domains. Consequently, all parameters of grain sizes, shapes, and EBSD indexes have been calculated for each domain separately. Sample COL-DR01-18 is too altered to characterize grain sizes and shapes. The corrected diameter *d** is the diameter multiplied by 1.2 to correct for sectioning effects before applying the piezometer of Van der Wal et al.[32] (Methods). Abbreviations: olivine (OL) – orthopyroxene (OPX).
